# Red Quinoa Hydrolysates with Antioxidant Properties Improve Cardiovascular Health in Spontaneously Hypertensive Rats

**DOI:** 10.3390/antiox12061291

**Published:** 2023-06-16

**Authors:** Miguel López-Moreno, Estefanía Jiménez-Moreno, Antonio Márquez Gallego, Gema Vera Pasamontes, José Antonio Uranga Ocio, Marta Garcés-Rimón, Marta Miguel-Castro

**Affiliations:** 1 Instituto de Investigación en Ciencias de Alimentación (CIAL, CSIC-UAM), 28049 Madrid, Spain; miguel.lopez@ufv.es (M.L.-M.); marta.miguel@csic.es (M.M.-C.); 2 Grupo de Investigación en Biotecnología Alimentaria, Universidad Francisco de Vitoria, 28223 Madrid, Spain; 3 Departamento de Ciencias Básicas de la Salud, Universidad Rey Juan Carlos (URJC), 28933 Alcorcón, Spaingema.vera@urjc.es (G.V.P.); jose.uranga@urjc.es (J.A.U.O.); 4 Grupo de Investigación de Alto Rendimiento en Fisiopatología y Farmacología del Sistema Digestivo (NeuGut), Universidad Rey Juan Carlos de Madrid (URJC), 28933 Alcorcón, Spain; 5 Unidad Asociada I+D+i al Instituto de Química Médica (IQM), Consejo Superior de Investigación Científicas (CSIC), 28006 Madrid, Spain

**Keywords:** antioxidant properties, enzymatic hydrolysates, quinoa, oxidative stress

## Abstract

In recent years, quinoa has been postulated as an emerging crop for the production of functional foods. Quinoa has been used to obtain plant protein hydrolysates with in vitro biological activity. The aim of the present study was to evaluate the beneficial effect of red quinoa hydrolysate (QrH) on oxidative stress and cardiovascular health in an in vivo experimental model of hypertension (HTN) in the spontaneously hypertensive rat (SHR). The oral administration of QrH at 1000 mg/kg/day (QrHH) showed a significant reduction in SBP from baseline (−9.8 ± 4.5 mm Hg; *p* < 0.05) in SHR. The mechanical stimulation thresholds did not change during the study QrH groups, whereas in the case of SHR control and SHR vitamin C, a significant reduction was observed (*p* < 0.05). The SHR QrHH exhibited higher antioxidant capacity in the kidney than the other experimental groups (*p* < 0.05). The SHR QrHH group showed an increase in reduced glutathione levels in the liver compared to the SHR control group (*p* < 0.05). In relation to lipid peroxidation, SHR QrHH exhibited a significant decrease in plasma, kidney and heart malondialdehyde (MDA) values compared to the SHR control group (*p* < 0.05). The results obtained revealed the in vivo antioxidant effect of QrH and its ability to ameliorate HTN and its associated complications.

## 1. Introduction

**Figure 1:**
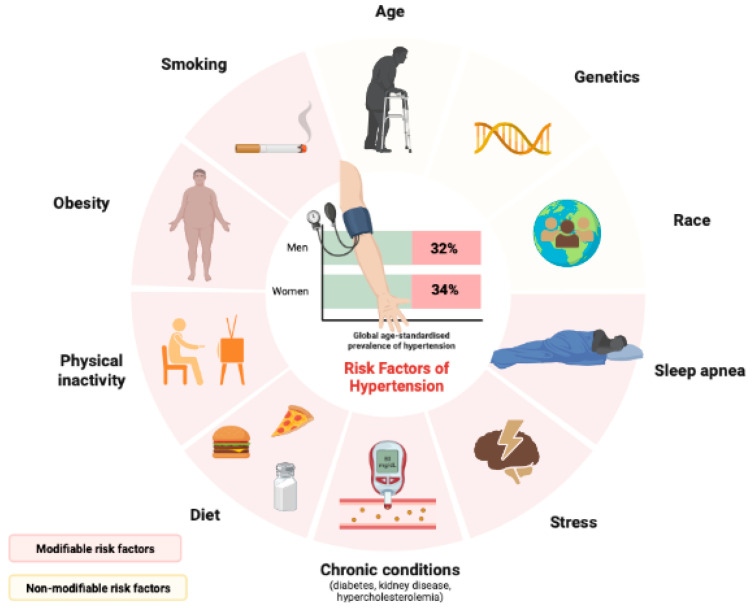
Main modifiable and non-modifiable risk factors that can increase the likelihood of developing CVDs and hypertension.

Hypertension is the leading cause of cardiovascular diseases (CVDs) and premature death worldwide. An estimated 1.28 billion adults aged 30–79 years suffer from hypertension worldwide (32% men and 34% women), with two thirds of whom living in low- and middle-income countries. Among individuals with hypertension, an estimated 46% are unaware that they have it. As a result, less than half of adults (42%) with hypertension are diagnosed and treated [1]. Its occurrence is multifactorial. There are several risk factors, many of them modifiable, that can increase the likelihood of developing CVDs and hypertension, and among them are the following: family history, age, race (higher risk in African-Americans), physical inactivity, sleep apnea, smoking and consumption of alcohol, stress, regular consumption of a diet rich in salt and fat, as well as chronic non-communicable diseases, such as diabetes, kidney disease, hyperlipidemia and, especially, obesity (Figure 1).

Some of the different modifiable risk factors include hypertension (HTN), hyperglycemia, hypercholesterolemia, obesity and smoking [2]. Among these factors, HTN represents the most important modifiable factor, accounting for 33% and 45.2% of the population’s attributable risk for CVDs and cerebrovascular disease, respectively [3,4]. The relevance of this risk factor is particularly important, as each 20 mm Hg increase in systolic blood pressure (SBP) and 10 mm Hg increase in diastolic blood pressure (DBP) is associated with a twofold increased risk of heart disease, cerebrovascular disease and other vascular pathologies [5]. Lifestyle modification is the first line of approach in the treatment of HTN [6]. However, when established lifestyle modifications fail to reduce blood pressure to the appropriate range for the patient, pharmacological treatment becomes of therapeutic relevance. Pharmacological interventions currently used for the treatment of HTN include classical drugs such as diuretics and beta-blockers, and newer drugs such as calcium channel blockers, angiotensin-converting enzyme inhibitors (ACE inhibitors) and angiotensin II receptor antagonists (ARBs) [7]. Among the main limitations of antihypertensive treatment is poor adherence to treatment, which has a negative impact on disease progression. In particular, it has been estimated that up to 45% of patients do not adhere satisfactorily to prescribed drug regimens [8].

The etiopathogenesis of HTN has been closely linked to oxidative stress. In particular, an excessive production and accumulation of reactive oxygen species (ROS) causes interactions with cellular structures, such as the modification of nucleic acid bases leading to mutagenesis and carcinogenesis [9]. They also alter protein structures through inactivation and denaturation, and act on lipids causing lipid peroxidation, which has the effect of altering the integrity and functionality of the membranes of biological systems [10]. Among these alterations, lipid peroxidation stands out, a chain reaction that triggers the formation of reactive compounds such as malondialdehyde (MDA) or 4-hydroxynonenal (HNE), which cross-link with proteins to form advanced lipoxidation products [11]. These compounds activate a cascade reaction that promotes the expression of pro-inflammatory cytokines and growth factors associated with the progression of different pathological conditions, including HTN [12].

Currently, there is growing interest in the research of food-derived antioxidant ingredients for the treatment of oxidative-stress-related diseases, such as HTN. These compounds include bioactive peptides, which are defined as inactive amino acid sequences present in the native protein, which are released after a process of enzymatic hydrolysis, fermentation or gastrointestinal digestion [13]. These bioactive peptides can exert different biological activities in the body with beneficial effects on human health and disease prevention [14]. Among them, antioxidant and antihypertensive food peptides have been the most investigated. Moreover, the growing trend toward plant-based diets has promoted the use of plant-based proteins as a source of bioactive peptides, due to their antioxidant properties, low cost, high biological activity and easy absorption [15].

In recent years, pseudocereals, and especially quinoa, have been used to obtain plant protein hydrolysates with biological activity [16,17,18]. Specifically, bioactive peptides derived from quinoa hydrolysis have been shown to exert antihypertensive activity through the inhibition of the angiotensin-converting enzyme (ACE) and antidiabetic activity mediated via the inhibition of dipeptidyl peptidase-4 (DPP-4) and alpha-glucosidase [18]. Similarly, different studies have shown improvements in the techno-functional properties of quinoa as a result of the enzymatic hydrolysis process [19]. In previous studies, we developed a red quinoa hydrolysate (QrH) with pleasant sensory properties, and demonstrated its in vitro antioxidant activity and in vivo antioxidant potential using Saccharomyces cerevisiae BY4741 (S. cerevisiae) as model organism (unpublished results); so, we speculate that the intake of QrH could be of interest in the management of arterial HTN. The aim of the present study was to evaluate the beneficial effect of QrH on oxidative stress and cardiovascular health in an in vivo experimental model of HTN in the spontaneously hypertensive rat (SHR).

## 2. Materials and Methods

### 2.1. Preparation of Quinoa Hydrolysate

Red quinoa was purchased from Naturquinoa (Madrid, Spain). The seeds were ground in a stone mill (Conasi, Jaén, Spain) and the flour was diluted in distilled water in a ratio of 1:12.5 (*w*/*v*). Enzymatic hydrolysis was carried out for 2 h with Alcalase 2.4 LFG (E.C. 3.4.21.61, from *Bacillus licheniformis*) provided by Novozymes (Copenhagen, Denmark), according to the manufacturer’s instructions. The hydrolysates were centrifugated at 4500 rpm for 15 min, and the supernatants were stored at −20 °C [20].

### 2.2. General Protocol in Spontaneously Hypertensive Rats

The experiments were designed to minimize the number of animals used and performed in accordance with the European and Spanish legislation on the care and use of experimental animals (210/63/UE; Real Decreto 53/2013), and were approved by the Ethics Committee at the University Rey Juan Carlos (URJC) (PROEX 174.7/21).

Thirty-two 10–11-week-old SHR males weighing 230–280 g and twenty-four 10–11-week-old WKY male rats weighing 230–280 g were purchased from Charles River Laboratories (Barcelona, Spain). The animals were housed in groups of four rats with 12 h light/dark cycles, at a temperature of 23 °C, and with ad libitum access to a standard laboratory rat diet, containing 0.1% sodium. After acclimatization, the rats were randomly divided into 7 groups (4 SHR and 3 WKY) and, for 8 weeks, until the rats were 20 weeks old, the drinking fluids in these groups were as follows: SHR control (tap water; *n* = 8), SHR vitamin C (a vitamin C solution 250 mg/kg/day; *n* = 8), SHR QrHL (a QrH solution 250 mg/kg/day; *n* = 8) and SHR QrHH (a QrH solution 1000 mg/kg/day; *n* = 8). In the case of WKY rats, the groups were as follows: WKY control (tap water; *n* = 8), WKY vitamin C (a vitamin C solution 250 mg/kg/day; *n* = 8) and WKY QrHH (a QrH solution 1000 mg/kg/day; *n* = 8). The daily doses used in this study for vitamin C and QrH were selected according to the results obtained in previous studies with vitamin C or other dietary protein hydrolysates [21,22,23,24].

During the experimental period, the body weight of the animals was recorded weekly until the end of the study. Daily water intake and food intake of the different groups were also estimated weekly. The occurrence of the neuropathic sign (tactile allodynia) was assessed once every 4 weeks using the Von Frey hair test [25]. The systolic blood pressure (SBP) of the rats was measured at the beginning and at the end of the study using the tail-cuff method.

At the end of the experimental period, at 20 weeks of age and after overnight fasting, the rats were anaesthetized with an intraperitoneal injection of ketamine (87 mg/kg) and xilacin (13 mg/kg) and euthanized by decapitation. Blood was collected in tubes containing lithium heparin as an anticoagulant. These samples were centrifuged at 500 G for 20 min at 4 °C to obtain plasma, which was divided into aliquots and kept frozen at −80 °C until analysis. Brain, heart, lungs, kidney, aorta, epididymal adipose tissue, liver and tibia were immediately excised and their weight recorded and kept frozen at −80 °C until analysis.

### 2.3. Systolic Blood Pressure Measurement

SBP was measured weekly using a standard non-invasive tail-cuff plethysmography method (Cibertec, Barcelona, Spain) in conscious, restrained rats, as described [23]. Briefly, rats were warmed to 37–38 °C for 10 min to detect caudal artery pulsations. Then, the transducer was placed on the tail, and the mean of the ten SBP determinations was used. To ensure the reliability of the measurements, previously, all animals were acclimatized, and measurements were performed by the same person at the same time of day.

### 2.4. Somatic Sensitivity: Von Frey Hair Test

The Von Frey test was performed to estimate mechanical sensitivity to non-noxious mechanical stimuli. In this test, a significant decrease in Von Frey, the hair withdrawal threshold evoked by tactile–mechanical stimuli, is suggestive of mechanical allodynia (increased sensitivity to non-noxious tactile stimuli). Mechanical sensitivity was assessed at weeks 0, 2, 6 and 10. Rats were individually placed on elevated iron mesh in a clear plastic cage and were allowed to adapt to the test environment for at least 10 min. Habituation to this environment was also performed the day before assessment. Von Frey calibrated hairs ranging from 4 to 26 g (4, 8, 10, 15 and 26 g) were applied to the plantar aspect of each hind paw, from below the mesh floor. This protocol was repeated five times with 3 s intervals. The withdrawal responses to the stimulus were recorded. A positive result was considered when at least three of five responses were obtained with each filament, and this value was considered to be the tactile threshold. When less than three positive responses were detected with any of the hair trials, the process was repeated with the next higher-force hair [24].

### 2.5. Oxidative Stress Biomarkers

Antioxidant capacity: Antioxidant activity in plasma and tissues (liver, heart, kidney and aorta) was determined using the oxygen radical absorbance capacity (ORAC) assay, as previously reported by Ou et al. (2001), with subsequent modifications [25,26]. ORAC values were quantified using a fluorimeter (FLUOstar Optima, BMG Labtech GmbH, Ortenberg, Germany) with wavelength excitation at 485 nm and wavelength emission measured at 520 nm. Results were expressed as µmol of trolox (Sigma-Aldrich, Saint-Louis, MO, USA) equivalent/µL of plasma and µmol of trolox equivalent/g of protein for liver, heart, kidney and aorta. The concentration of the protein present in each sample was determined using the Lowry method [27] modified by Peterson (1979) [28], using the Biorad diagnostic assay (DC protein assay, Biorad, Hercules, CA, USA) and bovine serum albumin as the standard.

Liver glutathione determination: Reduced glutathione (GSH) levels were determined via the monochlorobimane fluorimetric method, as previously described by Kamencic et al. (2000) [29], using a microplate reader (Infinite M200, Tecan US, Research Triangle Park, Morrisville, NC, USA) with wavelength excitation at 390 nm and wavelength emission measured at 510 nm. Results were expressed as µmol GSH/g protein.

Malondialdhehyde: Levels of malondialdehyde (MDA) in plasma and tissues (liver, heart, kidney and aorta) were measured via the thiobarbituric acid assay at 535 nm, using a microplate reader (Cytation 5 Biotek, Winooski, VT, USA) as previously described Manso et al. (2008). Results were expressed as nmol MDA/mL plasma and µmol of trolox equivalent/g of protein for liver, heart, kidney and aorta.

### 2.6. Histopathological Analysis

Tissues were fixed in buffered 10% formalin and embedded in paraffin. Tissues were cut in sections of 5 μm and stained with hematoxylin-eosin (HE) for general analysis. They were studied under a Zeiss Axioskop 2 microscope (Carl Zeiss Vision International GmbH, Aalen, Gemany) equipped with the image analysis software package LASX 3.7 (Leica AG, Heerbrugg, Switzerland). Qualitative analysis was performed on 2 to 4 slices of each tissue per animal under 5×–20× objectives.

### 2.7. Statistical Analysis

The results were expressed as mean values ± standard error of the mean (SEM) for a minimum of 6 rats, and analyzed utilizing Student’s t test and one- or two-way analysis of variance (ANOVA), using the GraphPad Prism 8 software (GraphPad Software, Inc., San Diego, CA, USA). Differences between the groups were assessed using the Bonferroni post hoc test. Statistically significant differences were considered for values of *p* < 0.05.

## 3. Results and Discussion

### 3.1. Effect of Red Quinoa Hydrolysate Administration on Body Weight and Food and Water Intake

**Figure 2:**
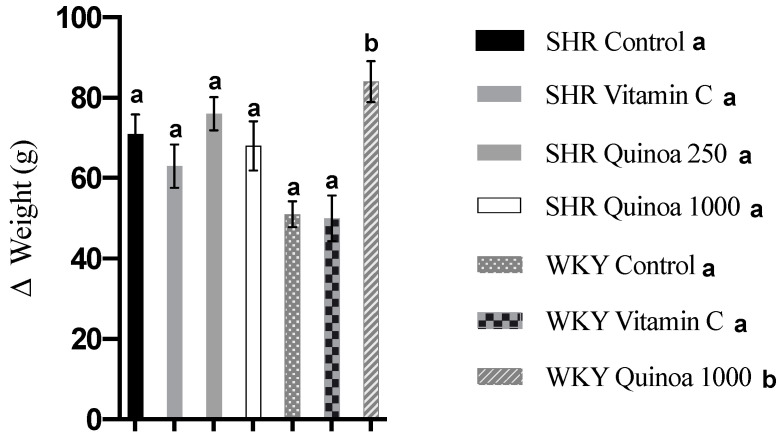
Body weight gain at the end of the treatment. Data represent mean values ± standard error of the mean (SEM) for each group. One-way ANOVA followed by Bonferroni post hoc test. Different letters indicate significant differences (*p* < 0.05).

In relation to body weight, the SHR and WKY rats showed a progressive increase in body weight from the beginning of the experimental period until the end of the study. WKY QrHH significantly increased their body weight compared to the control WKY rats (84 g vs. 51 g, *p* < 0.05). This trend was not observed in SHR, and body weight gain was similar in the groups that were given red quinoa hydrolysate and in the control group (Figure 2).

Regarding food intake, no significant differences were observed between the WKY and SHR throughout the study, nor between the different experimental groups of each of the strains of animals studied. In general, all animals increased their food intake from the beginning of the study until week 5, at which point food intake was stabilized in all experimental groups, with an average intake of 450–550 g/week. This estimation of weekly solid intake assumes a daily intake of 19–24 g/rat/day, which is within the usual range of solid intake of 14–35 g/day according to the literature [30,31].

**Figure 3:**
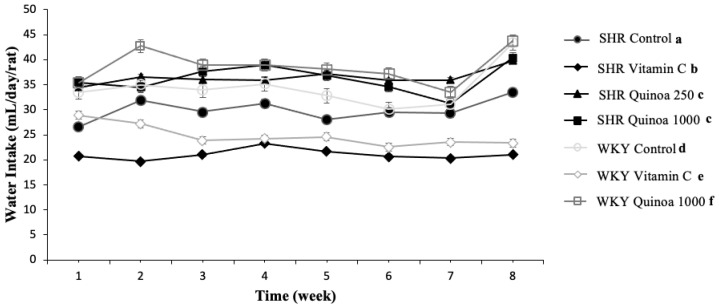
Evolution of water intake in the different experimental groups during the study. Data represent mean values ± standard error of the mean (SEM) for each group. Two-way ANOVA followed by Bonferroni post hoc test. Different letters indicate significant differences between groups (*p* < 0.05).

In terms of water intake, consumption was relatively stable throughout the study in the different groups, although there were differences in consumption between the groups (Figure 3). The water intake of both the WKY and SHR groups treated with red quinoa hydrolysate was significantly higher than their respective control groups (*p* < 0.05). In general, hydrolysates obtained from food proteins are often characterized by bitter tastes, which could negatively affect their intake [32,33]. However, the QrH used in this study exhibited adequate organoleptic characteristics, which could explain the higher liquid intake observed in the group treated with quinoa hydrolysate, as it was more palatable. According to the literature, it has been described that rats have an aversion to certain tastes, including acidic and bitter, and a greater predilection for sweet or salty tastes [34].

### 3.2. Effect of Red Quinoa Hydrolysate Administration on Blood Pressure

**Figure 4:**
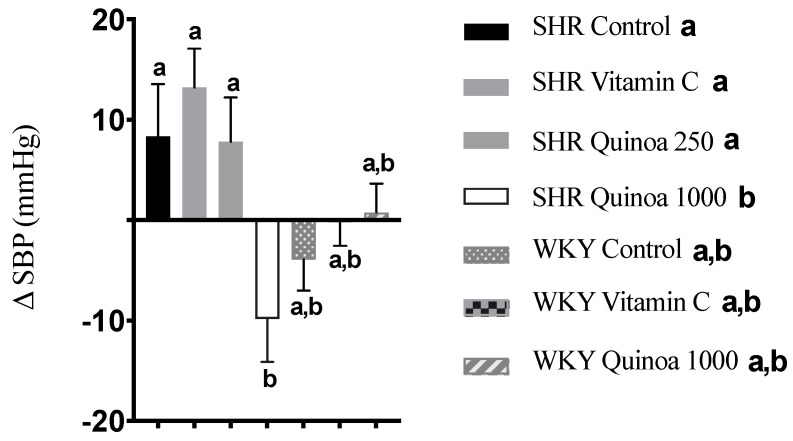
Increase in systolic blood pressure (SBP) in the different experimental groups during the 8-week experimental period. Data represent mean values ± standard error of the mean (SEM) for each group. One-way ANOVA followed by Bonferroni post hoc test. Different letters indicate significant differences between groups (*p* < 0.05).

SHR QrHH showed a significant reduction in SBP from baseline (−9.8 ± 4.5 mm Hg; *p* < 0.05), while no significant differences were found in the other SHR groups after 8 weeks of intervention (Figure 4). Bioactive peptides obtained from different plant-based matrices have also shown potential benefits on different parameters of cardiovascular health, including blood pressure modulation [35]. Rice bran protein hydrolysate has been shown to acutely reduce both SBP and diastolic blood pressure (DBP), similar to that observed for the reference antihypertensive compound captopril [36]. Ramírez-Torres et al. (2017) found that an amaranth protein hydrolysate with alcalase, the same enzyme selected in our study, at a dose of 1.2 g/kg, was able to induce a reduction in SBP in SHR 3 h after the ingestion of the hydrolysate to a magnitude similar to that reported for captopril [37]. The antihypertensive effect of quinoa protein hydrolysates has also been tested, and a reduction in blood pressure was observed between 2 and 6 h after the oral administration of different treatments. These antihypertensive effects were associated with changes in the microbiota, in particular with an increase in the abundance of the bacterial genera *Akkermansia*, *Turibacter* or *Allobaculum* [38]. These bacteria are involved in the production of metabolites such as short-chain fatty acids (SCFAs), which contribute to blood pressure regulation via immune modulation or the inhibition of angiotensin-converting enzyme (ACE) activity [39]. Similarly, a hydrolysate of quinoa proteins with pepsin and pancreatin, simulating gastrointestinal digestion conditions, resulted in a reduction in blood pressure between 2 and 10 h after oral administration [40]. Furthermore, the hydrolysate exhibited greater ACE inhibition than the control, suggesting that the bioactive peptides produced during hydrolysis may use this mechanism to exert their antihypertensive effect. According to the literature, cooked quinoa has been shown to increase levels of superoxide dismutase and catalase, key enzymes in the detoxification of ROS, in rats with HTN [41]. Therefore, it seems reasonable to suggest that the release of antioxidant peptides present in QrH could further enhance these effects on the different enzymes involved in redox balance.

### 3.3. Effect of Red Quinoa Hydrolysate Administration on Tactile Sensitivity

**Figure 5:**
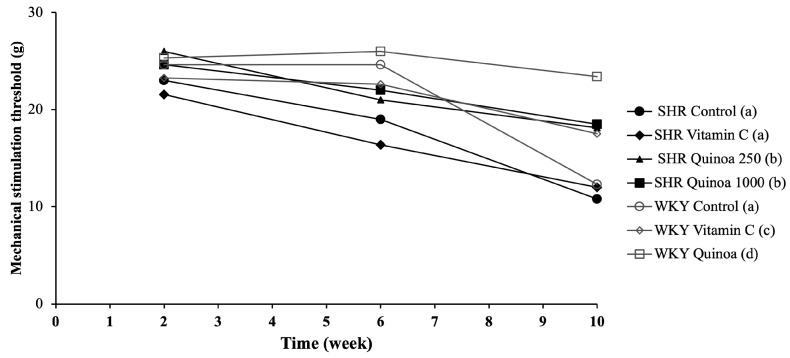
Mechanical stimulation threshold at weeks 2, 6 and 10 of the study, using the Von Frey filament test. Data represent mean values ± standard error of the mean (SEM) for each group. Two-way ANOVA followed by Bonferroni post hoc test. Different letters indicate significant differences between groups (*p* < 0.05).

The different groups of WKY rats maintained a very stable sensitivity threshold until week 6 of the study, and after which a significant decrease in the mechanical stimulation threshold was observed in the WKY control compared to the WKY vitamin C and WKY QrHH (*p* < 0.05) (Figure 5). This situation reveals the development of allodynia, the perception of pain that causes the paws to withdraw in response to a non-noxious tactile stimulus. In the SHR control, the threshold of mechanical sensitivity decreased throughout the study and markedly between week 6 and 10, being significantly lower than that reported in the WKY control (*p* < 0.05). This decreased mechanical threshold has been reported in other HTN models, such as salt-sensitive Dahl rats [42]. This model developed allodynia, hyperalgesia, neuroinflammation and increased oxidative stress [42]. Additionally, in SHR, thermal hyperalgesia has been previously reported [43].

In SHR QrHL, the sensitivity threshold decreased, but to a significantly lesser degree than the SHR control and SHR vitamin C (*p* < 0.05). In contrast, in SHR QrHH, the mechanical stimulation thresholds did not change throughout the study and remained constant throughout the treatment period. These findings indicate that the administration of QrH could be useful in the prevention of disorders linked to cutaneous sensitivity alterations, such as mechanical allodynia. Moreover, this effect is dose-dependent, since, as the dose of hydrolysate administered increases, the sensitivity threshold does not decrease. Free radicals cause the deterioration of peripheral nerves and induce a state of overexcitation of afferent nociceptors, which may contribute to the expression of neuropathic pain [44]. This situation may justify the usefulness of antioxidant compounds such as quinoa hydrolysate in neuropathic pain through the reduction in excessive reactive oxygen species production [45,46]. Other hydrolysates of different protein matrices such as egg white hydrolysates have been shown to be effective in reducing the risk of allodynia in different experimental models, and these results have been linked to antioxidant and inflammatory mechanisms [25,47,48,49]. Therefore, the results obtained regarding mechanical sensitivity in the rats of both groups, WKY and SHR, treated with the higher dose of QrH may be a consequence, at least in part, of the reduction in oxidative stress due to its antioxidant effect.

### 3.4. Effect of Red Quinoa Hydrolysate Administration on Organ Weight and Histology

Overall, the organs analyzed showed similar weights in the WKY and SHR groups regardless of the treatment administered, except for the liver (Table 1). SHR QrHH presented a lower relative liver weight compared to their control group (*p* < 0.05). In SHR, higher blood pressure has been associated with an increase in the liver/body weight ratio [50]. This situation could be due to the fact that oxidative stress is closely linked to HTN, which in turn induces structural and functional alterations at the hepatic level [51]. Antioxidant compounds such as resveratrol have demonstrated the ability to reduce MDA levels related to lipid peroxidation and increase the antioxidant capacity of liver tissue, ultimately leading to an improvement in liver tissue structural damage [52]. In our study, the differences observed in relative liver weight in SHR could suggest the ability of QrH to attenuate the functional changes that occur in this tissue that are associated with the development of HTN.

No changes in the microscopic structure of the arteries were observed. On the contrary, heart samples from SHR showed some hypereosinophilia, which is compatible with early cardiomyopathy. This is something that has been previously described, accompanied by cardiac fibrosis in aged SHR [53]. In our case, we did not observe fibrosis or variations in the groups treated with vitamin C or QrH, which can be explained by the fact that these were young animals where cardiac damage was not yet significant. We observed some glomerular atrophy, although not very extensive, which correlates with early hypertensive damage. Therefore, the results obtained suggest the potential usefulness of quinoa hydrolysate in improving the defense mechanisms against acute renal alterations caused by oxidative stress linked to HTN. Despite the described changes in the relative liver weight of the SHR QrHH group, no histological variations were observed in this tissue.

### 3.5. Effect of Red Quinoa Hydrolysate Administration on Oxidative Stress

**Figure 6:**
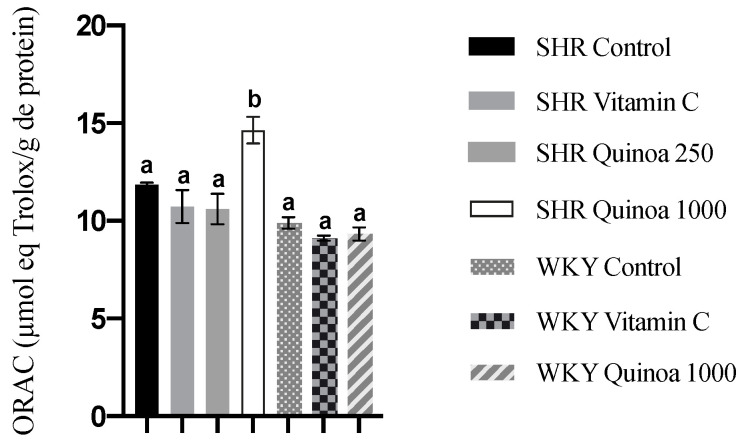
Antioxidant capacity of kidney assessed by oxygen radical absorbance capacity (ORAC). Data represent mean values ± standard error of the mean (SEM) for each group. One-way ANOVA followed by Bonferroni post hoc test. Different letters indicate significant differences between groups (*p* < 0.05).

In relation to antioxidant capacity in plasma, heart and aorta, the groups of both strains treated with QrH did not exhibit a significant increase in ORAC activity compared to their respective control groups (results not shown). Regarding the liver, no microscopic damage was observed. However, WKY vitamin C showed an increased antioxidant capacity compared to the control group (*p* < 0.05), while the QrH did not lead to significant changes in WKY or in SHR (results not shown). In turn, in the kidney, SHR QrHH exhibited a higher antioxidant capacity than the other experimental groups (*p* < 0.05) (Figure 6). The kidney is a target organ in HTN, and the initial vascular damage causes compensatory hypertrophy at the renal level. This situation triggers the collapse of some glomeruli, leading to hyperfiltration to maintain renal homeostasis and, finally, to proteinuria and glomerulosclerosis. These events are likely to occur between 30 and 60 weeks of age in the SHR [54]. Other antioxidant compounds have also been shown to increase intracellular antioxidant capacity at the renal level. Garcinia kola flavonoids have been shown to increase kidney ORAC values in a diabetic rat model by increasing the concentration of reduced glutathione [55].

**Figure 7:**
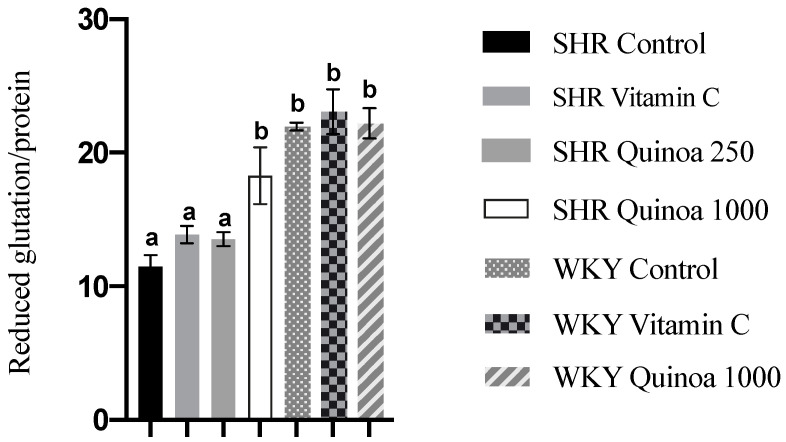
Reduced glutathione levels in the liver of the different experimental groups. Data represent mean values ± standard error of the mean (SEM) for each group. One-way ANOVA followed by Bonferroni post hoc test. Different letters indicate significant differences between groups (*p* < 0.05).

As observed in Figure 7, the SHR QrHH group showed an increase in reduced glutathione levels in the liver compared to the SHR control group. The consumption of QrH, due to its antioxidant character, and in a sufficient dose, could neutralize ROS and induce an increase in reduced glutathione biosynthesis. This increase in reduced glutathione levels in the liver has also been observed after the administration of protein hydrolysates from other food matrices [56]. Bioactive peptides present in a rice bran hydrolysate have been shown to activate nuclear factor erythroid 2-related factor 2 (Nrf2), which is involved in the regulation of reduced glutathione synthesis and proper redox balance [57]. Furthermore, according to the literature, antioxidant peptides may also be involved in restoring the activity of glutathione reductase, inhibited under oxidative stress conditions [58].

**Figure 8:**
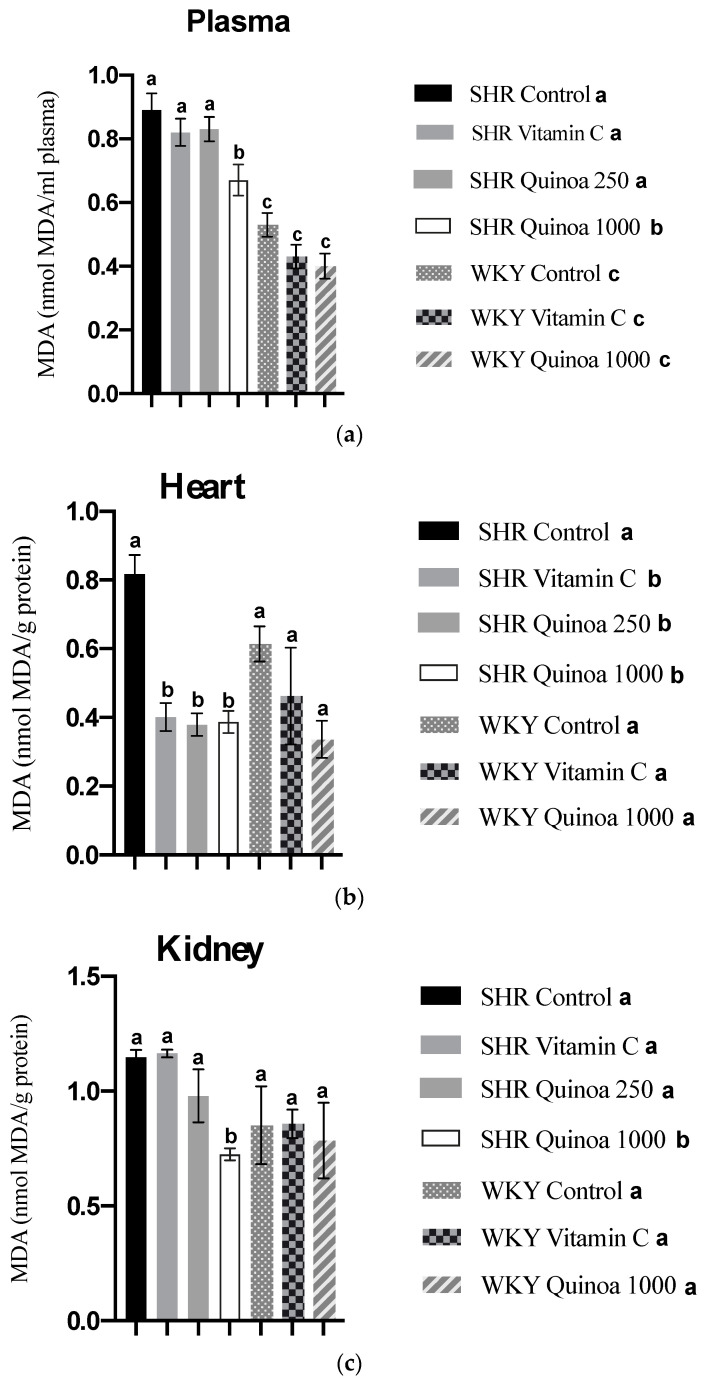
Malondialdehyde (MDA) concentration in plasma (**a**), heart (**b**) and kidney (**c**) at the end of the experimental period. Data represent mean values ± standard error of the mean (SEM) for each group. One-way ANOVA followed by Bonferroni post hoc test. Different letters indicate significant differences between groups (*p* < 0.05).

In relation to lipid peroxidation, the SHR QrHH group plasma MDA values decreased compared to the SHR control group (*p* < 0.05) (Figure 8a). These findings can be explained, at least in part, by the increased antioxidant activity at the plasma level observed in this group; although these differences were not significant compared to the SHR control, the observed trend highlights the possibility that this pathway was involved in the inhibition of lipid peroxidation. Protein hydrolysates have been shown in animal models of oxidative stress to reduce nitric oxide and MDA levels associated with increased plasma total antioxidant activity [59,60]. Likewise, SHR treated with vitamin C and doses of quinoa hydrolysate also showed a significant reduction in MDA levels in the heart (*p* < 0.05) (Figure 8b). According to the literature, different protein hydrolysates have been shown to reduce cardiac lipid peroxidation [61]. The effects of QrH could be related to the reduction in blood pressure observed in this study, considering that hypertensive patients commonly show increased levels of MDA at the systemic level [62]. It has been postulated that some bioactive compounds present in plant-based foods could be involved in the induction of antioxidant enzymes, such as superoxide dismutase and catalase, and through this mechanism act to decrease MDA levels and, therefore, oxidative stress [63]. On the other hand, QrH did not cause a significant change in liver and aortic MDA levels in SHR (results not shown).

Regarding lipid peroxidation in the kidney, SHR QrHH exhibited a significant decrease in kidney MDA levels compared to the other experimental groups (Figure 8c). Taking into consideration the ORAC results previously described, the increased antioxidant capacity observed in the kidney of SHR QrHH could counteract the formation of MDA and, thus, the development of lipid peroxidation in this target organ of HTN. These results are in line with previous studies that have reported the ability of bioactive peptides to reduce the concentration of MDA in the kidney [64,65]. Fish viscera hydrolysate has been proven to reduce kidney MDA levels in an experimental model of hypertension, similar to the reduction in MDA levels observed with captopril [66]. Among the mechanisms of action, it has been postulated that bioactive peptides could reduce MDA levels via the activation of enzymes involved in oxidative stress defense pathways, such as superoxide dismutase, glutathione peroxidase and catalase [65]. The antioxidant effect of HGR found in the kidney, the target organ of ETS, could be related to the structural/functional changes identified in this tissue.

## 4. Conclusions

Overall, the results obtained revealed the in vivo antioxidant effect of QrH and its ability to ameliorate HTN and its associated complications. The protective effect against oxidative stress exerted by QrH may be related to the antioxidant effect that the hydrolysate has demonstrated in previous studies in vitro. Further exploration of the mechanism of action of the hydrolysate is needed to elucidate the signaling pathways responsible for the effect of QrH on oxidative stress associated with HTN. In this regard, we are currently conducting studies to further evaluate its relationship with antioxidant enzyme production, lipid peroxidation and anti-inflammatory effects. These findings suggest an interest in conducting experimental studies with quinoa hydrolysates that will ultimately allow these products to be marketed as functional ingredients in gluten- and lactose-free foods or beverages with an appropriate sensory profile, and as being useful against pathologies related to oxidative stress, such as hypertension. This will require intervention studies to demonstrate their efficacy and safety in humans.

## Figures and Tables

**Table 1:** Relative organ weights obtained from rats at the end of treatment. Data represent the mean values (g) ± SEM for each group. Two-way ANOVA followed by Bonferroni post hoc test. Different letters indicate significant differences between groups.

Experimental Groups Organ Weight (g)	SHR Control	SHR Vitamin C	SHR Quinoa 250	SHR Quinoa 1000	WKY Control	WKY Vitamin C	WKY Quinoa 1000
Brain (g)	0.69 ± 0.02	0.61 ± 0.01	0.63 ± 0.03	0.64 ± 0.02	0.71 ± 0.02	0.71 ± 0.02	0.66 ± 0.02
Heart (g)	0.43 ± 0.02	0.44 ± 0.02	0.40 ± 0.01	0.44 ± 0.02	0.41 ± 0.02	0.37 ± 0.01	0.38 ± 0.01
Lungs (g)	0.52± 0.03	0.46 ± 0.03	0.54 ± 0.03	0.50 ± 0.03	0.72 ± 0.03	0.48 ± 0.02	0.52 ± 0.03
Liver (g)	3.35 ± 0.16 ^a^	3.13 ± 0.19 ^a^	2.95 ± 0.12 ^ab^	2.91 ± 0.12 ^b^	2.94 ± 0.13 ^a^	2.95 ± 0.14 ^a^	2.86 ± 0.09 ^a^
Spleen (g)	0.22 ± 0.01	0.19 ± 0.01	0.20 ± 0.01	0.22 ± 0.02	0.24 ± 0.02	0.22 ± 0.01	0.21 ± 0.01
Kidney (g)	0.81 ± 0.04	0.74 ± 0.04	0.74 ± 0.05	0.78 ± 0.05	0.69 ± 0.11	0.78 ± 0.03	0.69 ± 0.09
Epididymal adipose tissue (g)	1.33 ± 0.05	1.22 ± 0.08	1.29 ± 0.07	1.40 ± 0.06	1.41 ± 0.06	1.33 ± 0.07	1.42 ± 0.04
Muscle (g)	0.11 ± 0.03	0.05 ± 0.01	0.07 ± 0.02	0.06 ± 0.01	0.06 ± 0.01	0.05 ± 0.00	0.05 ± 0.00
Tibia (g)	1.13 ± 0.02	1.16 ± 0.03	1.11 ± 0.02	1.14 ± 0.04	1.09 ± 0.06	1.16 ± 0.03	1.09 ± 0.03

## Data Availability

Not applicable.
